# Dissipation Kinetics and Risk Assessment of Diniconazole, Dinotefuran, Metconazole, and Tebuconazole in *Raphanus sativus* L.

**DOI:** 10.3390/foods12152846

**Published:** 2023-07-27

**Authors:** Yunseon Kwak, Min-Ho Song, Ji-Woo Yu, Ji-Ho Lee

**Affiliations:** 1 Hazardous Substances Analysis Division, Gyeongin Regional Office of Food and Drug Safety, Incheon 22133, Republic of Korea; 2 Department of Crop Sciences, Konkuk University, Seoul 05029, Republic of Korea

**Keywords:** pesticides, dissipation pattern, risk assessment, *Raphanus sativus*, radish

## Abstract

This study investigated the degradation characteristics and conducted a risk assessment of four pesticides (Diniconazole, Dinotefuran, Metconazole, and Tebuconazole) in the leaves and roots of radish. Radish was cultivated in two greenhouse fields, and samples were collected at 0, 1, 2, 3, 5, 7, and 10 days after pesticide application. Sample analysis was performed using LC-MS/MS, and the recovery rates ranged from 70.1% to 118.6%. The biological half-life of Diniconazole was found to be 6.2 days (leaf and root), Dinotefuran was 5.3 days (leaf) and 4.6 days (root), Metconazole was 9.3 days (leaf) and 3.2 days (root), and Tebuconazole was 8.0 days (leaf) and 5.1 days (root). After comparing the maximum residue limits (MRL) of each pesticide in Korea with the residues during the pre-harvest interval (PHI), Diniconazole showed a Hazard quotient (HQ) exceeding 1, indicating potential risks for true consumers. Furthermore, Tebuconazole showed an HQ of 0.3 or higher, indicating a significant level of risk.

## 1. Introduction

In modern society, pesticides are considered economically crucial inputs in agriculture. They play a vital role in enhancing crop productivity and reducing time and labor costs, primarily through the advancement of herbicides [1,2]. However, the residual pesticides in agricultural products due to their use are a major factor in endangering human health [3]. Therefore, in the Republic of Korea (ROK), for agricultural products without set maximum residue limits (MRLs), the Positive List System (PLS) is applied to restrict pesticides to below 0.01 mg/kg as regulated by the Rural Development Administration (RDA) [4]. Additionally, to minimize damages to producers and consumers, ROK sets pre-harvest residue limits (PHRLs) to manage residual pesticides before cultivation. The PHRLs are determined and established using residual pesticide reduction constants and biological half-lives, and they predict residual amounts at harvest to prevent unsuitability resulting from exceeding MRLs [5]. Therefore, establishing MRLs and PHRLs for domestic agricultural products is essential to ensure safety.

Radish (*Raphanus sativus* L.) is an agricultural product in the Brassicaceae family, and it is consumed not only for its root but also for its leaves and sprouts. Radish contains various phytochemical contents such as flavonoids, terpenes, and minerals [6].

Dinotefuran (DNT) is a neonicotinoid insecticide that acts as a postsynaptic nicotinic receptor agonist, enhancing the activity of nicotinic acetylcholine receptors and resulting in insecticidal effects (Table S1) [7,8]. Diniconazole (DNC), metconazole (MTC), and tebuconazole (TBC) are triazole fungicides that act on cyp51 in the cytochrome P450 family, inhibiting the biosynthesis of ergosterol in fungi and causing fungicidal effects (Table S1) [9]. Although MRLs have been set for both radish leaves and roots in ROK [10], they have not been set in the EU [11]. In comparison to other countries, radish consumption through kimchi is relatively high in Korea. As a result of the high consumption of radish through kimchi in Korea, the potential harmful effects are also evaluated more critically. Therefore, the four pesticides used in this study were selected because they are commonly used in radish cultivation.

This study aimed to conduct the risk assessment of four pesticides in radish based on the dissipation kinetic data. In this study, we developed and validated a pesticide analysis method using HPLC-MS/MS with QuEChERS sample preparation for one neonicotinoid insecticide (DNT) and three azole fungicides (DNC, MTC, and TBC) in radish. Additionally, residue levels were determined on different days after the application of neonicotinoid insecticides and azole fungicides during radish cultivation to determine their half-lives and secure fundamental data for establishing PHRLs for residual pesticides in the production phase.

## 2. Materials and Methods

### 2.1. Materials and Standards

The standard pesticides (DNC, DNT, MTC, and TBC) were purchased from Sigma Aldrich (PO, USA). The purities of the standard pesticides ranged from 98.0% to 99.8%. The pesticides used in the packaging test, including Diniconazole 5% wettable powder (Vinnari), Dinotefuran 10% wettable powder (Osine), Metconazole 20% suspension concentrate (Sallimkkun), and Tebuconazole 20% suspension concentrate (Silvaco Plus), were purchased as commercial products. HPLC-grade Acetonitrile was purchased from Fisher Scientific (Seoul, Korea), and water was purchased from J.T. Baker (Phillipsburg, NJ USA). The solid reagent, formic acid, was purchased from Honeywell (Charlotte, NC, USA), and ammonium formate was purchased from Sigma Aldrich (Saint Louis, MO, USA). The QuEChERS extraction kit (EN 15662; MgSO_4_ 4 g, NaCl 1 g, Sodium citrate 1 g, Disodium citrate sesquihydrate 0.5 g) and QuEChERS dispersive kit (EN 15662; Magnesium sulfate 150 mg, PSA 25 mg) were purchased from CTK (Daejeon, Korea).

### 2.2. Greenhouse Experiments

Greenhouse experiments were conducted using cultivation sites located more than 20 km apart to account for geographical differences. The selected greenhouse facility sites were Eumseong, Chungcheongbuk-do (Field 1, 36.947427° N, 127.470263° E), and Icheon, Gyeonggi-do (Field 2, 37.309258° N, 127.406608° E). The experiments were carried out from March to April, and the radish variety used for all fields was Sangchun radish, which is generally cultivated in Korea and was purchased from a local farm (Table S2). Seeding was performed on March 8th for both Field 1 and Field 2. The experimental plots were selected with an area of 15 m^2^ per replicate, consisting of three replicates for the treatment group and one replicate for the control group. According to the pesticide safety guidelines, the formulated pesticide was diluted and prepared for application (Table 1). The formulation and method that achieve the maximum dispersion amount based on the active ingredient pesticides were selected for the spray formulation. It was processed following the safety guidelines for pesticide use as in RDA in ROK. After pesticide application, samples were collected at appropriate intervals for commercial harvest: immediately after treatment (within 2 h), 1, 2, 3, 5, 7, 10, and 14 days. The collected samples were chosen to ensure a combined weight of at least 1 kg, with a minimum of 12 individual samples. Each sample was labeled with information on the treatment and collection date, placed in a polyethylene bag, and stored in an icebox for transportation to the laboratory within 24 h. In the laboratory, the transported samples were divided into leaf and root parts, weighed for sample preparation, prepped, sectioned, and stored in a refrigerator (−20 °C or below) for at least 48 h. They were then homogenized using dry ice and a homogenizer and subsequently kept in frozen storage (−20 °C or below) until further analysis.

### 2.3. Standard Solution

Standard stock solutions of the four pesticides were prepared at a concentration of 1000 mg/L in acetonitrile. Working solutions at concentrations of 0.005, 0.01, 0.02, 0.05, 0.1, 0.2, and 0.5 mg/L were prepared by serially diluting the stock solutions with acetonitrile. Each of the working solutions was mixed with the extract of radish leaf or root in a 1:1 ratio to prepare the matrix-matched standard solutions of 0.005, 0.01, 0.02, 0.05, 0.1, and 0.25 mg/L.

### 2.4. Method Validation

Recovery tests were performed for all four pesticides at three different concentration levels: 0.01 mg/kg, 0.1 mg/kg, and the highest residue level for each pesticide. The tests were conducted with three replicates for each concentration level. Standard solutions were prepared to achieve concentrations of 0.01 mg/kg, 0.1 mg/kg, and the highest residue level in 10 g of the sample. Acetonitrile (10 mL) was added to the sample, and the mixture was shaken for 1 min. QuEChERS extraction kits (4 g MgSO_4_, 1 g NaCl, 1 g Na-Citrate, 0.5 g Disodium Citrate Sesquihydrate) were added to the extract, followed by shaking. The mixture was then centrifuged at 4000 rpm for 10 min. A total of 1 mL of the supernatant was transferred to a QuEChERS dispersive kit (150 mg MgSO_4_, 25 mg PSA), vortexed for 1 min, and then centrifuged at 15,000 rpm for 5 min. The supernatant of the resulting sample was filtered through a PTFE syringe filter (0.2 μm, 15 mm). After matrix matching performed by combining 100 μL of the filtrate with 100 μL of acetonitrile, the sample was injected into the LC-MS/MS system for quantitative analysis. The instrumental limits of quantification (ILOQ) for DNC, DNT, MTC, and TBC in radish were established as the minimum concentration of the analyte yielding signal-to-noise ratios greater than 10. The method limits of quantification (MLOQ) for the analytes were established by calculated using Equation (1).

(1) MLOQ(mg/kg)=ILOQ(mg/L)×[Final sample volume(mL)/Sample amount(g)]× Matrix-matched statndard dilution factor

### 2.5. LC-MS/MS Analytical Conditions

An AB Sciex Exion LC (Japan) liquid chromatograph equipped with a Tandem Mass spectrometer API 3200 (Japan) detector, coupled with an Acquity UPLC^®^ BEH Shield RP18 1.7 μm (2.1 × 100 mm column), was used for the analysis of the studied pesticides. The column oven temperature was maintained at 40 °C, and the injection volume was 2 μL. The mobile phases consisted of water with 0.1% formic acid and 5 mM ammonium formate (A), and methanol with 0.1% formic acid and 5 mM ammonium formate (B). The flow rate was 0.2 mL/min. The gradient conditions were set as follows: 0 min, B = 20%; 1 min, B = 20%; 3 min, B = 100%; 8 min, B = 100%; 9 min, B = 20%; 10 min, B = 20%. The following MS instrumental conditions with a positive ESI source were applied: an ion spray voltage of 5500 V, nitrogen as the collision gas, and a capillary temperature of 400 °C. Table 2 provides the pesticides’ MRM transitions and MS conditions.

### 2.6. Storage Stability and Daily Residue Levels

For storage stability, untreated samples of 10 g were prepared with DNC, DNT, MTC, and TBC standard solutions at a concentration of 10 times the limit of quantification (LOQ) (0.10 mg/kg). The samples were thoroughly mixed to achieve uniformity and then stored in a freezer at temperatures below −20 °C. After a storage period of at least 150 days for both above-ground and below-ground parts, the samples were analyzed using the same method as the recovery test to determine the residue levels.

For the analysis of daily residue levels of DNC, DNT, MTC, and TBC during the cultivation period of radish, the samples were processed and analyzed using the same method as the recovery test.

### 2.7. Statistical Analysis

The determination of daily residue levels of DNC, DNT, MTC, and TBC in radish was performed using regression analysis. Firstly, the residue decay constants and biological half-lives were calculated using Equations (2) and (3). Secondly, F-test and *t*-test were conducted to verify the significance of the regression equations and decay constants. The lower limit of the decay constant at a 95% confidence level was determined. This analysis was carried out using the regression analysis test table provided by the Ministry of Food and Drug Safety. Based on the residue limits of DNC, DNT, MTC, and TBC in radish leaves and roots, daily residue levels were estimated until 10 days before harvest to derive the PHRL for the harvest stage [12,13].

(2) $$C_{t}=C_{0}\times e^{-kt}$$

(3) $$t_{1/2}=\ln\times 2/k,$$

*C*_0_ represents the initial residue concentration (mg/kg) of the pesticide, indicating the residue level at the last sampling point, which is 2 h after the final application. *C_t_* represents the residue concentration (mg/kg) at time *t*, which indicates the number of days after the last spray. *k* represents the constant dissipation rate.

### 2.8. Dietary Risk Assessment

A dietary risk assessment was conducted for the residues of DNC, DNT, MTC, and TBC in radish. The estimated daily intake (EDI) was calculated using the residue levels on day 0, day 7, or day 14 of radish consumption, considering the radish intake and the MRLs of the pesticides. Furthermore, the hazard quotient (HQ) was determined using the acceptable daily intake (ADI) and utilized in the hazard assessment [12,14,15] (Equations (4) and (5)).

(4) $$\mathrm{EDI}\;=C_{t}\times \;\mathrm{IR}/\mathrm{BW}$$

(5) HQ= EDI/ADI,

EDI, which stands for estimated daily intake (mg/kg b.w./day), represents the estimated amount of pesticide intake per kilogram of body weight per day. In Korea, it is consumed both raw and processed. According to reference report, both radish leaves and root were more eaten raw than processed, and we used EDI considering both raw and processing factor for leaves and root [16]. *C_t_* refers to the residue concentration of pesticides in radish leaves or roots (mg/kg), while IR represents the average daily intake of radish leaves or roots (kg/day) obtained from the Standardization Guidelines for Estimating Food Intake conducted in 2019 by the Ministry of Food and Drug Safety, involving 43,602 individuals. The average consumption of radish leaves and roots was found to be 0.00651 kg and 0.02047 kg per person per day, respectively [16]. Among the total survey respondents (*n* = 43,602), there were 3098 individuals (7.11%) and 21,581 individuals (49.50%) who were true consumers of radish leaves and roots, respectively. The average daily intake of radish leaves and roots among true consumers (*n* = 3098 and 21,581) was determined to be 0.09317 kg and 0.04040 kg per person per day, respectively. Body weight data were obtained from the 5th and 6th Korean National Health and Nutrition Examination Survey (KNHNES) [17], with an average weight of 59.44 kg among the survey participants. The acceptable daily intake (ADI) values for DNC, DNT, MTC, and TBC are 0.0023, 0.02, 0.04, and 0.03 mg/kg b.w./day, respectively [10]. Values below 1 indicate that the population is not at risk of exposure. All statistical data for this study were obtained from a publicly accessible database.

## 3. Results and Discussion

### 3.1. Method Validation

**Figure 1:**
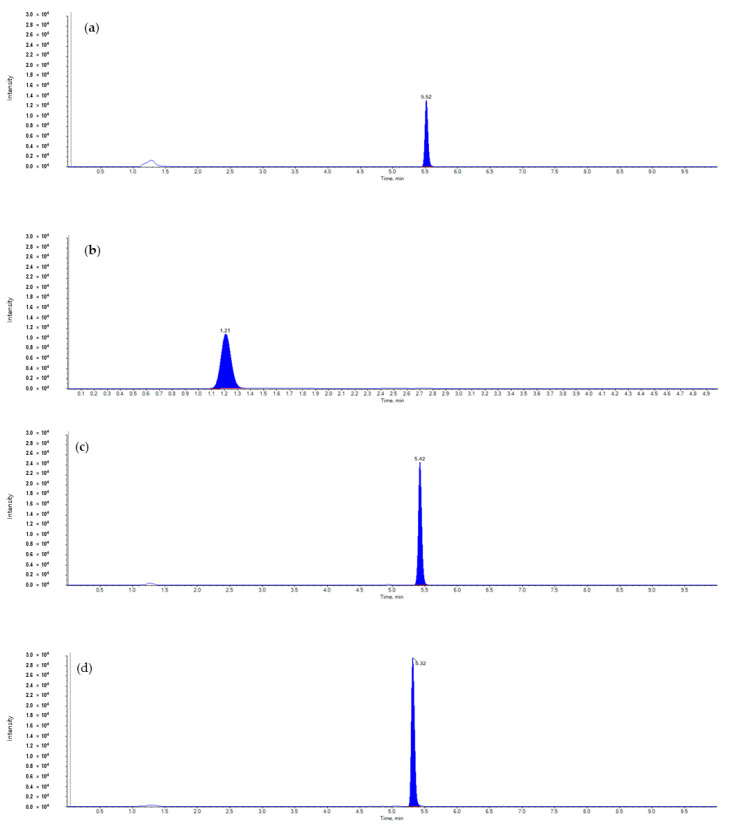
Representative chromatogram (0.1 mg/kg) of (**a**) diniconazole, (**b**) dinotefuran, (**c**) metconazole, (**d**) tebuconazole.

The ILOQ for pesticides in radish were 0.005 mg/L and MLOQ were 0.01 mg/kg. The calibration curves of the standard solutions were established through regression analysis of peak area within the concentration range, and the linearity within the concentration range was confirmed by the determination coefficient (r^2^) (Table 3). The regression equations obtained from the regression analysis are presented in Table 4. The recovery tests were conducted with three replicates from each field. The results of the recovery tests at 0.01 mg/kg, 0.1 mg/kg, and the highest concentration were all within the acceptable criteria for method validation in residue analysis during the production phase, which are recovery rates of 70–110% and a coefficient of variation within 20% (Table 4). During the quantification analysis using LC-MS/MS for DNC, DNT, MTC, and TBC in radish, it was confirmed that there was no interference from other peaks in both the untreated samples and the recovery samples (Figure 1). The storage stability test at a concentration of 0.1 mg/kg showed recovery rates of 80–110% for all components, indicating that there was no decomposition or loss during the sample storage period.

### 3.2. Daily Residue Levels

In the leaf part of radish, the initial residue levels of TBC were approximately 2.1 times, 2.1 times, and 1.7 times higher on average compared to DNC, DNT, and MTC. In the root part of radish, the initial residue levels of DNT were approximately 2.4 times, 1.1 times, and 1.4 times higher on average compared to DNC, MTC, and TBC (Table 5).

During spraying, the recommended safe usage of DNC was three applications within seven days prior to harvest, DNT was three applications within 14 days prior to harvest, MTC was one application within 14 days prior to harvest, and TBC was two applications within seven days prior to harvest. Accordingly, the recommended dilution rates for spray solutions were as follows: DNC wettable powder at a dilution rate of 2000-fold with a 5% concentration, DNT wettable powder at a dilution rate of 1000-fold with a 10% concentration, MTC suspension concentrate at a dilution rate of 3000-fold with a 20% concentration, and TBC suspension concentrate at a dilution rate of 2000-fold with a 20% concentration. Based on this, the total amount of effective ingredient applied via spraying was found to be four times higher for DNT compared to DNC, 4.5 times higher compared to MTC, and 2.25 times higher compared to TBC.

The residue levels relative to the total amount of effective ingredient applied are shown in Table 1. In the leaves, MTC was 1.4 times higher than DNC, 5.6 times higher than DNT, and 1.2 times higher than TBC. In the roots, MTC was 2.4 times higher than DNC, 4.0 times higher than DNT, and 2.5 times higher than TBC. The reasons for the differences in pesticide degradation despite the TSA are generally attributed to various factors such as the physicochemical properties of the pesticide, formulation, cultivation environment, and crop cultivation characteristics [18,19,20].

### 3.3. Residual Characteristics of Pesticides in Radish Leaf and Root

The transitions in residue levels over time following the application of DNC, DNT, MTC, and TBC during the cultivation period of radish were investigated. Regression analysis was conducted based on the residue data obtained on different harvest dates to determine residue dissipation patterns and rate constants.

**Figure 2:**
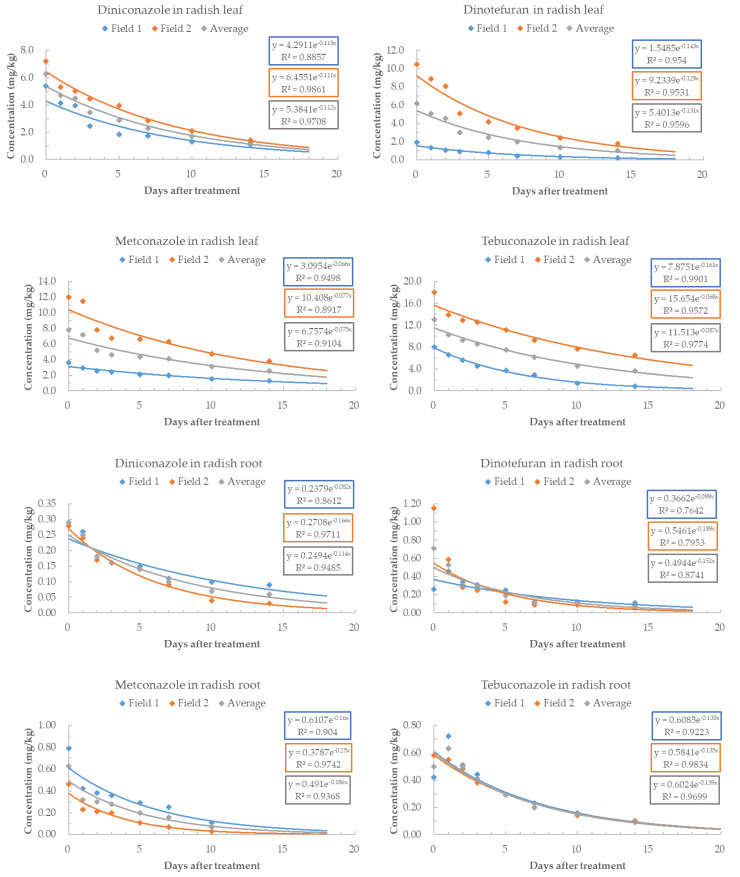
Dissipation patterns of pesticides in radish leaf and root.

During the cultivation period of radish, both in the leaf and root parts, DNC, MTC, and TBC showed exponential decline in residue levels. On the 14th day, which was the final harvest day, the initial residue levels of DNC were 20.2% in Field 1 (leaf), 31.0% in Field 1 (root), 19.4% in Field 2 (leaf), and 10.7% in Field 2 (root). For DNT, the corresponding values were 12.9% (leaf) in Field 1, 42.3% (root) in Field 1, 16.6% (leaf) in Field 2, and 5.1% (root) in Field 2. MTC showed residue levels below 1.3% (leaf) in Field 1, below 2.2% (root) in Field 1, below 36.6% (leaf) in Field 2, and below 31.6% (root) in Field 2. TBC exhibited residue levels of 10.3% (leaf) in Field 1, 21.4% (root) in Field 1, 35.9% (leaf) in Field 2, and 17.2% (root) in Field 2 (Figure 2).

The pre-harvest interval (PHI) for radish was 7 days for DNC and TBC, and 14 days for DNT and MTC. On the respective harvest dates, the residue levels in the leaf and root parts were as follows: DNC 2.28 mg/kg, 0.10 mg/kg (average of Field 1 and 2), DNT 1.00 mg/kg, 0.08 mg/kg (average of Field 1 and 2), MTC 2.55 mg/kg, 0.00 mg/kg (average of Field 1 and 2), and TBC 6.11 mg/kg, 0.20 mg/kg (average of Field 1 and 2). Comparing these residue levels with the MRLs for the respective pesticides, DNC exceeded the limits in the leaf part (2.0 mg/kg) and root part (0.05 mg/kg), DNT exceeded the limit in the root part (0.05 mg/kg), TBC exceeded the limits in the leaf part (5.0 mg/kg) and root part (0.2 mg/kg), while DNT met the limit in the leaf part (1.5 mg/kg), and MTC met the limits in the leaf part (20 mg/kg) and root part (0.05 mg/kg), as per the corresponding MRLs.

Simple regression analysis was used to determine the regression equations for residue levels in different parts of radish on a daily basis (Figure 2). The coefficients of determination (r^2^) were all above 0.87, indicating high correlation and explanatory power. The biological half-lives of pesticides in different parts of radish are presented in Table 6. For DNC, the geometric mean of the biological half-life from previous studies was 4.25 days [21], with a half-life of 2.9–3.1 days in tea leaves [22]. Comparing the half-life of DNC in radish leaves with that in other leaves, radish leaves tend to persist for a longer period of time than others. The biological half-life of DNT was 10.06 days (geometric mean) [21], 10 days in spinach leaves [23], and 4.65–5.5 days in tea leaves [24]. The half-life of DNT in radish leaves was found to be similar to tea leaves. MTC exhibited a half-life of 6.9 days in perilla leaves [25]. Indeed, perilla leaves have a shorter half-life compared to radish leaves. The half-life of TBC was predicted to have a geometric mean half-life of 7.67 days [21], 9.4–9.9 days in green onion leaves [26], 5.11–7.22 days in ginseng leaves [27], 1.85–2.46 days in wheat leaves [28], 2.22–2.23 days in spinach leaves [29], 6.4 days in perilla leaves [25], and 11.6–25.3 days in bell pepper leaves [30]. In the case of roots, the biological half-life of TBC in ginseng was 4.59–5.46 days [27]. Radish leaves have a longer half-life compared to other leaves in the previous studies, with the exception of bell pepper leaves, which have the longest half-life. In the case of radish roots, they exhibit a similar pattern to the results in ginseng.

### 3.4. Calculation of Maximum Residue Limits during the Harvest Stage of Radish

The MRLs during the harvest stage are established to ensure that pesticide residue levels at the time of crop harvest do not exceed the specified limits. These limits are determined by applying the lower bound of the 95% confidence interval of the regression coefficient for residue levels at a specific time point before harvest. The lower bounds of the 95% confidence interval for the rate constants were determined as follows: DNC leaf part 0.0929 and root part 0.0837 (average of Field 1 and 2), DNT leaf part 0.1044 and root part 0.0905 (average of Field 1 and 2), MTC leaf part 0.0513 and root part 0.1639 (average of Field 1 and 2), and TBC leaf part 0.0738 and root part 0.1121 (average of Field 1 and 2). Based on these values, the calculated MRLs are presented in Table 7.

The MRLs for radish 10 days before harvest were determined as follows: DNC leaf part 5.06 and root part 0.12 mg/kg, DNT leaf part 4.26 and root part 0.12 mg/kg, MTC leaf part 33.42 and root part 0.26 mg/kg, and TBC leaf part 10.46 and root part 0.61 mg/kg.

### 3.5. Dietary Risk Assessment

Hazard assessment was performed for the residues of DNC, DNT, MTC, and TBC in radish. If the HQ value exceeds 1, it indicates a risk of exposure to the pesticide. Table 8 presents the results of the hazard assessment for DNC, DNT, MTC, and TBC on day 0, day 7, and day 14 of radish consumption (average of Field 1 and 2) among the entire surveyed population and actual consumers. When considering the entire population, the highest hazard was observed for DNC in radish leaves on day 0, with a value of 3.00 × 10^−1^. For radish roots, all HQ values from day 0 to day 14 were below 1.09 × 10^−1^, indicating a relatively low hazard.

However, considering the significant difference in consumption between actual consumers and the total average consumers, additional evaluations were conducted. For DNC, a HQ value of 4.29 was observed in leaf samples on day 0, exceeding 1, and a value of 8.57 × 10^−1^ was found in root samples, surpassing 0.8, indicating a very high hazard. Furthermore, on day 7, which corresponds to the PHI period, leaf samples also exhibited a HQ value exceeding 1, indicating a high hazard for actual consumers. In contrast, the other three pesticides showed very low hazards, with HQ values below 6.82 × 10^−1^ for all leaf and root samples on days 0, 7, and 14. Except for DNC during the PHI period, none of the three pesticides exceeded a HQ value of 1 in all parts of radish. These findings indicate that actual consumers of radish leaves and roots are at a significantly higher risk of exposure compared to the entire surveyed population, with leaf consumption posing a 14.31-fold higher risk and root consumption a 2.82-fold higher risk. In a previous study conducted on other crops where HQ assessments targeted actual consumers, high levels of toxicity were observed in the actual consumers, similar to the results of this study [31]. Additionally, according to the National Nutrition Statistics from the Korea Centers for Disease Control and Prevention, there is a noticeable increase in radish consumption per kilogram of body weight starting from the age of 30 [32]. Considering the daily consumption of radish leaves and roots, there is a higher risk of pesticide exposure for DNC and TBC during the PHI period, while the other pesticides seem to be safe.

## 4. Conclusions

In the greenhouse harvest stages of radish, we investigated the decline patterns and residue characteristics of the neonicotinoid insecticide Dinotefuran and the triazole fungicides Diniconazole, Metconazole, and Tebuconazole. Based on this assessment, we conducted a risk evaluation considering pesticide toxicity. The decline patterns of residues in the two fields exhibited differences in initial residue levels when applying the approach of total sprayed effective ingredient. Despite similar cultivation conditions, the pesticides showed different half-lives. The risk assessment, using the pesticide residue levels during the respective PHI periods, indicated that all HQ values for the overall survey participants were below 1, suggesting low risks. However, for actual consumers, Diniconazole exceeded an HQ of 1, indicating a very high risk, while Tebuconazole showed an HQ above 0.3, indicating a potential risk. These findings provide fundamental data for re-evaluating and establishing domestic standards such as MRLs, PHI, or PHRLs.

## Figures and Tables

**Table 1:** Types of pesticides and application details.

Pesticide	Formulation		Application				PHI (Days)
	Type	AI	Dilution Rate	Spray No.	Interval (Days)	TSA	
Diniconazole	WP	5	2000	3	7	0.2475	7
Dinotefuran	WP	10	1000	3	7	0.99	14
Metconazole	SC	20	3000	1	7	0.22	14
Tebuconazole	SC	20	3000	2	7	0.44	7

AI, Active ingredient, %, TSA, Total sprayed amount of pesticides = active ingredient content/dilution factor × application times × >application rate, g, PHI, Pre-harvest interval, WP, Wettable powder, SC, Suspension concentrate.

**Table 2:** Multiple reaction monitoring conditions of four pesticides.

Analytes	Ionization	Precursor Ion (*m/z*)	Product Ion (*m*/*z*)		Retention Time (min)
			Quantitation (Collision Energy, eV)	Qualification (Collision Energy, eV)	
Diniconazole	[M + H]^+^	326.1	70.2 (42)	159.1 (40)	5.5
Dinotefuran	[M + H]^+^	203.1	129.2 (17)	114.2 (17)	1.2
Metconazole	[M + H]^+^	320.0	70.0 (58)	125.0 (56)	5.4
Tebuconazole	[M + H]^+^	308.0	70.0 (51)	125.0 (49)	5.3

**Table 3:** Linear equation of the calibration curves for the quantification of the four pesticide residues in radish leaf and root.

Pesticide	Linear Range (mg/L)	Radish Leaf				Radish Root			
		Field 1		Field 2		Field 1		Field 2	
		Linear Equation	R^2^	Linear Equation	R^2^	Linear Equation	R^2^	Linear Equation	R^2^
DNC	0.005–0.25	y = 240376.5527x + 356.7611	0.9903	y = 242721.8089x − 83.5911	0.9908	y = 248804.1197x + 321.0432	0.9969	y = 244659.5860x + 591.6900	0.9950
DNT		y = 285107.3727x + 1330.1962	1.0000	y = 261336.3591x + 10356.9996	0.9993	y = 254921.7192x + 1574.7351	0.9988	y = 227456.5412x + 1764.5123	0.9966
MTC		y = 919210.5027x + 1209.6831	0.9997	y = 916146.4301x + 637.5441	0.9999	y = 837243.8481x + 871.9971	0.9997	y = 828347.2924x + 818.4145	0.9999
TBC		y = 1007501x + 1291.8580	0.9999	y = 1007846.6112x + 1287.1053	0.9997	y = 962329.3438x + 775.5557	0.9999	y = 1012285.6426x − 468.7282	0.9996

**Table 4:** Recovery tests and storage tests in radish leaf and root.

Pesticide	Spiking Level (mg/kg)		Recovery (%)								MLOQ (mg/kg)
			Radish Leaf				Radish Root				
			Field 1		Field 2		Field 1		Field 2		
			Average ± SD	CV	Average ± SD	CV	Average ± SD	CV	Average ± SD	CV	
DNC	Recovery	0.01	81.6 ± 2.8	3.5	87.3 ± 2.9	3.4	84.7 ± 2.5	2.9	73.5 ± 1.8	2.4	0.01
		0.10	87.5 ± 2.5	2.9	80.7 ± 1.9	2.4	96.3 ±. 1.3	1.4	84.3 ± 1.7	2.1	
		Highest	87.9 ± 2.8 ^(2)^	3.2	85.1 ± 4.0 ^(2)^	4.8	106.7 ± 2.1 ^(1)^	2.0	84.3 ± 1.7 ^(1)^	7.3	
	Storage	0.10	87.5 ± 1.2	1.4	81.1 ± 3.1	3.8	97.7 ± 1.4	1.4	85.8 ± 2.4	2.8	
DNT	Recovery	0.01	86.0 ± 11.8	13.8	83.2 ± 9.7	11.7	88.0 ± 12.5	14.2	83.7 ± 2.8	3.4	0.01
		0.10	103.7 ± 1.4	1.3	93.6 ± 1.5	1.6	114.0 ±. 4.4	3.8	115.8 ± 4.0	3.7	
		Highest	99.0 ± 7.7 ^(1)^	7.8	107.7 ± 4.0 ^(1)^	3.7	87.9 ± 1.9 ^(1)^	2.2	108.7 ± 4.0 ^(1)^	7.3	
	Storage	0.10	102.8 ± 4.6	1.4	99.2 ± 5.9	5.9	109.1 ± 2.9	2.7	100.5 ± 1.5	1.5	
MTC	Recovery	0.01	93.4 ± 1.1	1.2	91.3 ± 2.1	1.2	98.5 ± 1.9	2.9	97.4 ± 2.0	2.1	0.01
		0.10	98.7 ± 0.8	0.8	101.7 ± 0.9	0.8	101.6 ± 1.3	1.4	106.0 ± 3.6	3.4	
		Highest	100.8 ± 6.9 ^(1)^	6.9	112.9 ± 5.7 ^(1)^	6.9	97.1 ± 2.1 ^(1)^	2.0	96.4 ± 12.3 ^(1)^	12.8	
	Storage	0.10	87.5 ± 1.2	2.4	101.7 ± 0.8	0.8	100.1 ± 1.1	1.1	104.2 ± 3.3	3.2	
TBC	Recovery	0.01	87.5 ± 6.4	7.3	88.6 ± 8.3	9.3	95.9 ± 2.4	2.5	106.3 ± 2.8	2.6	0.01
		0.10	106.3 ± 2.5	2.4	107.2 ± 2.3	2.1	107.2 ± 1.5	14	101.4 ± 2.3	2.3	
		Highest	100.2 ± 5.8 ^(2)^	5.8	105.0 ± 6.3 ^(2)^	6.0	106.4 ± 8.4 ^(1)^	7.9	102.7 ± 11.0 ^(1)^	10.7	
	Storage	0.10	104.0 ± 2.1	2.1	104.7 ± 1.6	1.6	109.4 ± 1.2	1.1	102.5 ± 1.6	1.5	

SD, Standard deviation, CV, Coefficients of variation, MLOQ, Method limit of quantification, ^(1)^ 5 mg/kg spiking level, ^(2)^ 10 mg/kg spiking level.

**Table 5:** Multiple reaction monitoring conditions of four pesticides.

Pesticide	Harvest Time (after Spraying the Pesticides)	Radish Leaf		Radish Root		KOR MRL of Radish (mg/kg)	EU MRL of Radish (mg/kg)
		Field 1	Field 2	Field 1	Field 2		
DNC	0	5.39 ± 0.52	7.21 ± 0.61	0.29 ± 0.02	0.28 ± 0.01	2.0 (leaf) 0.05 (root)	-
	1	4.14 ± 0.09	5.31 ± 0.18	0.26 ± 0.02	0.24 ± 0.02		
	2	3.97 ± 0.10	5.03 ± 0.06	0.18 ± 0.01	0.17 ± 0.00		
	3	2.45 ± 0.08	4.47 ± 0.18	0.16 ± 0.00	0.16 ± 0.01		
	5	1.84 ± 0.28	3.96 ± 0.56	0.15 ± 0.00	0.14 ± 0.01		
	7	1.72 ± 0.26	2.85 ± 0.43	0.11 ± 0.02	0.09 ± 0.01		
	10	1.31 ± 0.13	2.08 ± 0.19	0.10 ± 0.00	0.04 ± 0.00		
	14	1.09 ± 0.07	1.40 ± 0.10	0.09 ± 0,00	0.03 ± 0.00		
DNT	0	1.94 ± 0.01	10.48 ± 0.39	0.26 ± 0.01	1.15 ± 0.06	1.5 (leaf) 0.05 (root)	-
	1	1.34 ± 0.14	8.85 ± 0.28	0.46 ± 0.02	0.59 ± 0.03		
	2	1.07 ± 0.04	8.05 ± 0.69	0.34 ± 0.04	0.28 ± 0.02		
	3	0.94 ± 0.04	5.07 ± 0.23	0.31 ± 0.03	0.25 ± 0.04		
	5	0.81 ± 0.05	4.16 ± 0.17	0.25 ± 0.02	0.12 ± 0.02		
	7	0.45 ± 0.03	3.50 ± 0.33	0.12 ± 0.02	0.09 ± 0.01		
	10	0.34 ± 0.04	2.40 ± 0.16	0.12 ± 0.00	0.09 ± 0.01		
	14	0.25 ± 0.04	1.75 ± 0.32	0.11 ± 0,01	0.06 ± 0.01		
MTC	0	3.58 ± 0.47	11.98 ±1.59	0.79 ± 0.11	0.46 ± 0.01	20.0 (leaf) 0.05 (root)	-
	1	2.89 ± 0.13	11.47 ± 1.77	0.42 ± 0.04	0.23 ± 0.01		
	2	2.56 ± 0.15	7.80 ± 13.41	0.38 ± 0.01	0.21 ± 0.00		
	3	2.40 ± 0.03	6.76 ± 2.10	0.36 ± 0.02	0.20 ± 0.01		
	5	2.08 ± 0.09	6.61 ±0.65	0.29 ± 0.02	0.11 ± 0.00		
	7	1.96 ± 0.06	6.27 ±0.05	0.25 ± 0.01	0.07 ± 0.01		
	10	1.54 ± 0.05	4.70 ±11.59	0.11 ± 0.00	0.03 ± 0.00		
	14	1.31 ± 0.25	3.78 ± 6.28	<0.01	<0.01		
TBC	0	8.04 ± 0.11	18.03 ± 0.70	0.42 ± 0.02	0.58 ± 0.05	5.0 (leaf) 0.2 (root)	-
	1	6.56 ± 0.51	13.93 ± 1.12	0.72 ± 0.11	0.55 ± 0.01		
	2	5.55 ± 0.40	12.93 ± 0.08	0.51 ± 0.00	0.48 ± 0.03		
	3	4.52 ± 0.21	12.93 ± 0.08	0.44 ± 0.04	0.38 ± 0.05		
	5	3.74 ± 0.32	11.15 ± 1.71	0.29 ± 0.01	0.29 ± 0.03		
	7	2.95 ± 0.37	9.27 ± 0.58	0.23 ± 0.01	0.20 ± 0.03		
	10	1.42 ± 0.19	7.69 ± 0.65	0.16 ± 0.03	0.14 ± 0.01		
	14	0.83 ± 0.08	6.47 ± 0.49	0.09 ± 0.01	0.10 ± 0.02		

**Table 6:** Half-lives of pesticides in radish leaves and roots (days).

Pesticide	Radish Leaf			Radish Root		
	Field 1	Field 2	Average	Field 1	Field 2	Average
DNC	6.1	6.2	6.2	8.6	4.1	6.2
DNT	4.9	5.4	5.3	6.9	3.6	4.6
MTC	10.5	9.0	9.3	4.4	2.7	3.2
TBC	4.3	10.2	8.0	5.2	5.0	5.1

**Table 7:** Recommended PHRLs for pesticides radish leaves and roots.

Pesticide		Recommended PHRLs (mg/kg)				MRLs (mg/kg)
		10 Days before	7 Days before	5 Days before	3 Days before	
DNC	Leaf	5.06	3.83	3.18	2.64	2.0
	Root	0.12	0.09	0.08	0.06	0.05
DNT	Leaf	4.26	3.11	2.53	2.05	1.5
	Root	0.12	0.09	0.08	0.07	0.05
MTC	Leaf	33.42	28.65	25.85	23.33	20.0
	Root	0.26	0.16	0.11	0.08	0.05
TBC	Leaf	10.46	8.38	7.23	6.24	5.0
	Root	0.61	0.44	0.35	0.28	0.2

**Table 8:** (**a**) HQ of four pesticides for total survey participants in relation to radish leaf and root consumption. (**b**) HQ of four pesticides for true consumers in relation to radish leaf and root consumption.

**(a)**						
**Pesticide**			**Residue Value (mg/kg)**	**ADI**	**EDI (mg/kg b.w./day)**	**HQ**
DNC	Day 0	Leaf	6.30	0.0023	6.90 × 10^−4^	3.00 × 10^−1^
		Root	0.29		9.99 × 10^−5^	4.34 × 10^−2^
	Day 7	Leaf	2.28		2.50 × 10^−4^	1.09 × 10^−1^
		Root	0.10		3.44 × 10^−5^	1.50 × 10^−2^
	Day 14	Leaf	1.25		1.37 × 10^−4^	5.95 × 10^−2^
		Root	0.06		2.07 × 10^−5^	8.98 × 10^−3^
DNT	Day 0	Leaf	6.21	0.02	6.80 × 10^−4^	3.40 × 10^−2^
		Root	0.71		2.45 × 10^−4^	1.22 × 10^−2^
	Day 7	Leaf	1.98		2.17 × 10^−4^	1.08 × 10^−2^
		Root	0.11		3.79 × 10^−5^	1.89 × 10^−3^
	Day 14	Leaf	1.00		1.10 × 10^−4^	5.48 × 10^−3^
		Root	0.08		2.76 × 10^−5^	1.38 × 10^−3^
MTC	Day 0	Leaf	7.78	0.04	8.52 × 10^−4^	2.13 × 10^−2^
		Root	0.35		1.21 × 10^−4^	3.01 × 10^−3^
	Day 7	Leaf	4.11		4.50 × 10^−4^	1.13 × 10^−2^
		Root	0.07		2.41 × 10^−5^	6.03 × 10^−4^
	Day 14	Leaf	2.55		2.79 × 10^−4^	6.98 × 10^−3^
		Root	<0.001		<3.44 × 10^−7^	<8.61 × 10^−6^
TBC	Day 0	Leaf	13.06	0.03	1.43 × 10^−3^	4.77 × 10^−2^
		Root	0.71		2.45 × 10^−4^	8.15 × 10^−3^
	Day 7	Leaf	6.11		6.69 × 10^−4^	2.23 × 10^−2^
		Root	0.20		6.89 × 10^−5^	2.30 × 10^−3^
	Day 14	Leaf	3.65		4.00 × 10^−4^	1.33 × 10^−2^
		Root	0.09		3.10 × 10^−5^	1.03 × 10^−3^
**(b)**						
**Pesticide**			**Residue Value (mg/kg)**	**ADI**	**EDI (mg/kg b.w./day)**	**HQ**
DNC	Day 0	Leaf	6.30	0.0023	9.88 × 10^−3^	4.29
		Root	0.29		1.97 × 10^−4^	8.57 × 10^−2^
	Day 7	Leaf	2.28		3.57 × 10^−3^	1.55
		Root	0.10		6.80 × 10^−5^	2.96 × 10^−2^
	Day 14	Leaf	1.25		1.96 × 10^−3^	8.52 × 10^−1^
		Root	0.06		4.08 × 10^−5^	1.77 × 10^−2^
DNT	Day 0	Leaf	6.21	0.02	9.73 × 10^−3^	4.87 × 10^−1^
		Root	0.71		4.83 × 10^−4^	2.41 × 10^−2^
	Day 7	Leaf	1.98		3.10 × 10^−3^	1.55 × 10^−1^
		Root	0.11		7.28 × 10^−5^	3.74 × 10^−3^
	Day 14	Leaf	1.00		1.57 × 10^−3^	7.84 × 10^−2^
		Root	0.08		5.44 × 10^−5^	2.72 × 10^−3^
MTC	Day 0	Leaf	7.78	0.04	1.22 × 10^−2^	3.05 × 10^−1^
		Root	0.35		2.38 × 10^−4^	5.95 × 10^−3^
	Day 7	Leaf	4.11		6.44 × 10^−3^	1.61 × 10^−1^
		Root	0.07		4.76 × 10^−5^	1.19 × 10^−3^
	Day 14	Leaf	2.55		4.00 × 10^−3^	9.99 × 10^−2^
		Root	<0.001		<6.80 × 10^−7^	<1.70 × 10^−5^
TBC	Day 0	Leaf	13.06	0.03	2.05 × 10^−2^	6.82 × 10^−1^
		Root	0.71		4.83 × 10^−4^	1.61 × 10^−2^
	Day 7	Leaf	6.11		9.58 × 10^−3^	3.19 × 10^−1^
		Root	0.20		1.36 × 10^−4^	4.53 × 10^−3^
	Day 14	Leaf	3.65		5.72 × 10^−3^	1.91 × 10^−1^
		Root	0.09		6.12 × 10^−5^	2.04 × 10^−3^

ADI, acceptable daily intake; EDI, estimated daily intake; HQ, hazard quotient.

## Data Availability

Data will be made available on request.

## Supplementary Materials

The following supporting information can be downloaded at: https://www.mdpi.com/article/10.3390/foods12152846/s1, Table S1: Chemical structures and physicochemical properties of the four pesticides; Table S2: Greenhouse air temperature and humidity date during cultivation of radish plants.
