# Supplementary material for: Dissipation Kinetics and Risk Assessment of Diniconazole, Dinotefuran, Metconazole, and Tebuconazole in Raphanus sativus L
Source: Foods. 2023 Jul 27;12(15):2846. doi: 10.3390/foods12152846 (PMC10417377; doi:10.3390/foods12152846)
Supplement: Supplementary file 1 [file foods-12-02846-s001.zip › foods-2499492-supplementary.pdf]

Table S1 Chemical structures and physicochemical properties of the four pesticides

Pesticide	Diniconazole	Dinotefuran	Metconazole	Tebuconazole
**Structure**	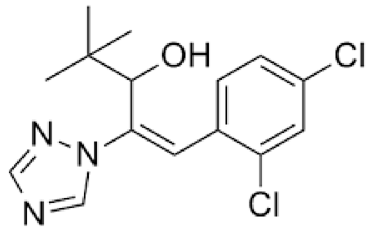	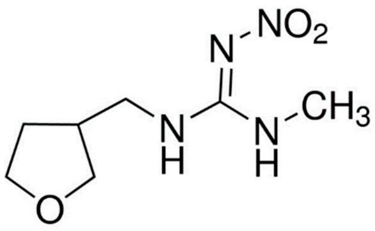	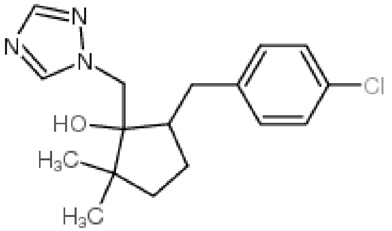	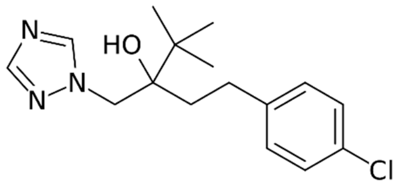
**IUPAC name**	(E)-1-(2,4-dichlorophenyl)-4,4-dimethyl-2-(1,2,4-triazol-1-yl)pent-1-en-3-ol	(RS)-1-methyl-2-nitro-3-(tetrahydro-3-furylmethyl)guanidine	5-[(4-chlorophenyl)methyl]-2,2-dimethyl-1-(1,2,4-triazol-1-ylmethyl)cyclopentan-1-ol	(RS)-1-p-chlorophenyl-4,4-dimethyl-3-(1H-1,2,4-triazol-1-ylmethyl)pentan-3-ol
**Appearance**	Colourless crystals	White crystalline solid	White crystalline solid	Colourless crystals; (tech., colourless to light brown powder)
**Vapor pressure**	2.93 mPa (20 °C); 4.9 mPa (25 °C)	<1.7 × 10-3 mPa (30 °C)	2.1 × 10-5 mPa (20 °C)	1.7 × 10-3 mPa (20℃) (OECD 104)
**Kow logP**	4.3 (25 °C)	-0.549 (25 °C)	3.85	3.7 (20℃)
**Solubility**	In water 4 mg/l (25 °C). In acetone, methanol 95, xylene 14, hexane 0.7 (all in g/kg, 25 °C).	In water 39.8 g/l (20 °C). In hexane 9.0 × 10-6, heptane 11 × 10-6, xylene 72 × 10-3, toluene 150 × 10-3, dichloromethane 11, acetone 58, methanol 57, ethanol 19, ethyl acetate 5.2 (all in g/l, 20 °C).	Water solubility 30.4 mg/l (20 °C) methanol(g/l, 20 °C) 403, acetone(g/l, 20 °C) 363	In water 36 mg/L (pH 5-9, 20℃). In dichloromethane>200, isopropanol, toluene 50-100, hexane<0.1(all in g/L, 20℃)

Table S2 Greenhouse air temperature and humidity date during cultivation of radish plants (a) Field 1, (b) Field 2

(a)						
**Date**	**Temperature (℃)**				**Day precipitation (mm)**	**Treatment**
	**Average**	**High**	**Low**	**Humidity(%)**		
2022-04-05	7.5	34.9	18.3375	50.98542		1st in 3 times every 7 days
2022-04-06	9.8	35.5	18.09375	50.30208		
2022-04-07	6.1	37	18.45	49.30833		
2022-04-08	9.6	37.9	20.05208	53.23333		
2022-04-09	8.5	39.9	21.16667	53.86042		
2022-04-10	11.7	40	22.69792	57.92708		
2022-04-11	13.1	30.8	21.13333	56.55833		
2022-04-12	9.8	17.9	14.10208	79.08333		2nd in 3 times every 7 days<br>1st in 2 times every 7 days
2022-04-13	9.5	22.4	13.51042	84.06042		
2022-04-14	4.7	27.7	14.00625	65.9875		
2022-04-15	4.8	23.7	12.97083	54.01667		
2022-04-16	3.7	28.2	14.90625	53.56458		
2022-04-17	6.7	26.4	15.17708	56.90208		
2022-04-18	4	28	14.61875	54.22083		
2022-04-19	3.8	30.5	15.05208	53.2125		3rd in 3 times every 7 days<br>2nd in 2 times every 7 days<br>1st in 1 times every 7 days<br>Day 0 harvesting
2022-04-20	4.5	25.1	14.26458	55.20833		Day 1 harvesting
2022-04-21	7.4	25.4	16.55833	61.64375		Day 2 harvesting
2022-04-22	8.7	31.7	18.41458	62.825		Day 3 harvesting
2022-04-23	9.2	30.5	18.73542	57.19583		
2022-04-24	14.5	28.9	20.4375	63.28333		Day 5 harvesting

2022-04-25	16.1	35.2	21.20208	67.86875		
2022-04-26	11.8	30.4	19.31042	67.39167		Day 7 harvesting
2022-04-27	10.5	31.4	18.72708	66.8125		
2022-04-28	12.7	20.5	15.97917	75.67708		
2022-04-29	11.2	22.7	15.16667	72.85417		Day 10 harvesting
2022-04-30	8.7	24.7	15.27292	64.84792		
2022-05-01	9.5	24.9	15.4625	63.66667		
2022-05-02	7	23.9	16.54375	56.50625		
2022-05-03	19.4	20.2	19.82708	39.4125		Day 14 harvesting

**(b)**						
**Date**	**Temperature (℃)**				**Day precipitation (mm)**	**Treatment**
	**Average**	**High**	**Low**	**Humidity(%)**		
2022-04-05	0.8	25.2	11.9125	49.7617		1st in 3 times every 7 days
2022-04-06	3.6	26.4	13.91042	52.66667		
2022-04-07	5.4	25.4	14.69792	54.98333		
2022-04-08	2	27.9	12.64167	50.3125		
2022-04-09	5.6	29.3	15.675	46.70625		
2022-04-10	5.1	32.4	17.41042	43.97021		
2022-04-11	7.5	32.7	18.83958	44.30851		
2022-04-12	11.7	33	20.60208	50.39375		2nd in 3 times every 7 days<br>1st in 2 times every 7 days
2022-04-13	8.8	16.9	11.6625	66.35417		
2022-04-14	7.4	15.4	10.02083	66.20833		
2022-04-15	1.5	24.7	12.65625	56.23261		
2022-04-16	1.7	24.7	12.15208	48.71489		
2022-04-17	0.4	27.6	13.5125	44.11277		

2022-04-18	7.1	28.1	14.675	42.46667		
2022-04-19	2.2	29.5	13.94583	41.60455		3rd in 3 times every 7 days<br>2nd in 2 times every 7 days<br>1st in 1 times every 7 days<br>Day 0 harvesting
2022-04-20	2	30.6	14.48958	42.06522		Day 1 harvesting
2022-04-21	3.5	27.7	14.4	44.41957		Day 2 harvesting
2022-04-22	7.3	25.9	14.67917	55.83333		Day 3 harvesting
2022-04-23	7.9	30.6	16.65	54.3413		
2022-04-24	8.7	33	18.95833	50.59111		Day 5 harvesting
2022-04-25	16	30.1	20.36042	59.10208		
2022-04-26	15.3	32.3	21.53542	60.15532		Day 7 harvesting
2022-04-27	10	30.7	18.65208	43.19756		
2022-04-28	6.6	32	17.89167	44.0186		
2022-04-29	9.8	21.3	14.14375	51.4		Day 10 harvesting
2022-04-30	8.4	24	13.84167	52.29111		
2022-05-01	2.6	27.1	13.9625	50.99792		
2022-05-02	4	26.8	13.51667	53.53125		
2022-05-03	2.1	30.5	17.71875	55.47234		Day 14 harvesting
